# Radiographic prevalence of juvenile osteochondral conditions of the proximal interphalangeal joint of Australian Thoroughbred racehorse yearlings and associations with sales results and race performance

**DOI:** 10.3389/fvets.2022.988826

**Published:** 2022-10-10

**Authors:** Josephine Faulkner, Katrien Vanderperren, Luc Duchateau, Chris O'Sullivan

**Affiliations:** ^1^Department of Morphology, Imaging, Orthopedics, Rehabilitation and Nutrition, Faculty of Veterinary Medicine, Ghent University, Merelbeke, Belgium; ^2^Biometrics Research Centre, Faculty of Veterinary Medicine, Ghent University, Merelbeke, Belgium; ^3^Randwick Equine Centre Equine Specialists, Horsley Park, NSW, Australia

**Keywords:** equine, radiography, proximal interphalangeal joint, subchondral lucency, osteochondral fragment

## Abstract

**Objectives:**

Ascertain the radiographic prevalence and variation in characteristics of juvenile osteochondral conditions (JOC) in the proximal interphalangeal joint (PIPJ) of Australian Thoroughbred racehorse yearlings. Establish whether there are any significant associations with public auction sale results and racing performance.

**Methods:**

Retrospective evaluation of 1,098 yearling repository radiograph sets. Comparison of sales results and whole career racing performance of the case group with two control groups: maternal siblings (*N* = 397) and yearlings without PIP JOC (*N* = 391).

**Results:**

6.3% of yearlings had at least one PIPJ JOC lesion with 4.8% having subchondral lucencies of the proximal phalanx (P1SC), 0.6% with subchondral lucencies of the middle phalanx (P2SC) and 0.8% with osteochondral fragmentation (OCF). P1SC were more prevalent in forelimbs and P2SC and OCF were more commonly located in the hindlimbs. 51% of PIPJ JOC were not identified on a lateromedial projection (LM). A significantly lower proportion of horses with OCF were successfully sold at public auction (*p* ≤ 0.05) but there was no significant difference in sales price between the case group and controls. A lower proportion of horses with PIPJ JOC made it to the racetrack to race, although this was not statistically significant. There was no significant difference in racing performance between the case group and controls, although there was a trend toward case horses earning lower career prize money and lower prize money per race (*p* ≤ 0.1). Lesions located in a dorsal or palmar/plantar location on the LM projection earned a lower average prize money per race (*p* ≤ 0.05) than those in a central location, and showed a trend toward earning lower total prize money (*p* ≤ 0.1) and number of places (*p* ≤ 0.1). There was no significant difference in performance for horses with lesions at the medial, axial or lateral aspects of the articular surface.

**Clinical importance:**

Overall, the findings of this study indicate that the presence of PIPJ JOC in radiographs of Thoroughbred yearlings should be attributed a low to moderate risk to future racing performance, however certain lesion characteristics may be associated with decreased performance.

## Introduction

Radiographic abnormalities of the proximal interphalangeal joint (PIPJ) have been described in young horses of numerous breeds including Thoroughbreds, Standardbreds, Quarter Horses and Warmbloods (1–10). Reported abnormalities predominantly comprise of juvenile osteochondral conditions (JOC) such as shallow indentations in subchondral bone contour, subchondral lucencies and osteochondral fragmentation however there are also descriptions of osteophytosis, osteoarthritis, juvenile degenerative joint disease, fractures, and axial palmar or plantar enthesophytes.

In comparison with other joints of the appendicular skeleton, there is a relative lack of information about the clinical relevance of lesions within the PIPJ of young horses and the potential impact on future performance. Although there are numerous studies of Thoroughbreds which have investigated the prevalence of articular radiographic abnormalities in yearling survey radiographs and any significant effects of the lesions on future racing performance, the proximal interphalangeal joint either did not feature in these studies (11–14) or the low numbers of cases with PIPJ lesions prevented specific analysis of the region (15).

Nonetheless, it has been shown that PIPJ JOC can be clinically relevant. A case series of young horses with osteochondral fragments of presumed developmental etiology at the dorsomedial aspect of the PIPJ had lameness attributed to the joint in 4/9 joints (7). A review of the radiographic reports of 10,169 weanlings and yearling in Kentucky, USA identified radiographic abnormalities of the pastern in 1.3% of horses (5). In this population, no lesion had a statistically significant effect on racing performance when compared with a control group comprised of maternal siblings. However horses in this study with fragments in the pastern tended to be less likely to start a race at 2 or 3 years old (*p* = 0.15). Horses with fragments or subchondral bone cysts in the distal aspect of the proximal phalanx tended to be less likely to start a race at 2 or 3 years of age than the control group (*p* = 0.08 and *p* = 0.17, respectively). Another Kentucky-based study presented as a conference abstract identified 171 yearlings from repository radiographs that had subchondral lucencies in the PIPJ and compared the racing performance with a maternal sibling control group (6). There was no significant difference in performance for those with lucencies in the medial or lateral aspects of the condyles, however those with midline lucencies had decreased starts as a 2-year-old as well as earnings and earnings per race during the 2 and 3-year-old racing seasons (all *p* ≤ 0.05).

The use of maternal siblings for the control group in these investigations may introduce certain confounding problems for statistical analysis since the prevalence of the same lesions is not determined in the control group. As genetics are a risk factor for the development of JOC it is possible that at least some of the matched sibling controls may also have had similar PIPJ lesions and therefore potentially diminishing differences between the two groups. Due to the contrasting results between these investigations and unknown prevalence of PIPJ lesions in the control groups, there is ongoing uncertainty of the level of risk to assign to lesions of the proximal interphalangeal joint in young Thoroughbred horses. Furthermore, no investigation has been performed with Thoroughbred horses outside of North America and Europe therefore it cannot be certain that the findings are transferable to other Thoroughbred populations.

The aims of this study were (1) to ascertain the radiographic prevalence of juvenile osteochondral conditions in the PIPJ of Australian Thoroughbred yearlings presented for sale, specifically osteochondral fragmentation and subchondral lucencies, (2) to describe their variability in size and location and (3) to determine if there were any significant associations of these lesions with sales price and future racing performance in comparison with horses without the lesions. Subchondral lucencies in the distal aspect of the proximal phalanx (P1) and the proximal aspect of the middle phalanx (P2) were hypothesized to have no effect on racing performance, regardless of location within the joint. Horses with osteochondral fragmentation were hypothesized to have a poorer racing performance. Sales results were predicted to parallel racing performance.

## Materials and methods

### Study population

The radiographic reports for all yearling Thoroughbreds undergoing pre-sales radiographic examination by Randwick Equine Center (REC), Australia between December 2007 and March 2011 were reviewed. The radiographs were all acquired for the official repository of public auction sales and were acquired within 42 days of the chosen auction. For pre-sale survey radiographic examinations of Thoroughbred yearlings in Australia it is recommended that all radiographic projections of the fetlocks should include the pastern joint (16). The compulsory projections of the metacarpophalangeal joint are the dorsopalmar elevated 20° (DPa), flexed lateromedial (flexed LM), dorso 45–55° lateral-palmaromedial oblique elevated 5–10° (DLPaMO) and dorso 45–55° medial-palmarolateral oblique elevated 5–10° (DMPaLO) projections. Compulsory projections of the metatarsophalangeal joint are the dorsoplantar elevated 30° (DPl), standing lateromedial (LM), dorso 45–55° lateral-plantaromedial oblique elevated 15° (DLPlMO) and dorso 45–55° medial-plantarolateral oblique elevated 15° (DMPlLO). The PIPJ is also visualized on the lateromedial projection of the forelimb feet. Radiographs of the hindlimb feet are not required by the repository.

A portable digital radiography unit (EKLIN EDR3 MkII with a CANON Gadox detector) and portable generator (MinXray HF8020) were used for radiographing the fetlocks and feet, with exposure factors of 74kVp and 1.2 mAs. Radiographs were originally read and reported on by one of three experienced equine veterinarians (>12 years in practice), including two specialist equine surgeons.

### Case selection

On retrospective review, all reports that noted the presence of osteochondral fragments or subchondral bone lucencies in the PIPJ in either the forelimbs or hindlimbs were short-listed. Terms leading to selection were “lucency,” “subchondral lucency,” “cyst,” “subchondral bone cyst,” “subchondral defect,” “concavity,” “fragment,” “fragmentation,” “osteochondral fragment,” and “osteochondral fragmentation.” Included locations were the distal aspect of the proximal phalanx or proximal aspect of the middle phalanx. Other radiographic abnormalities of the proximal phalangeal joint were not recorded and these joints were considered normal for the purpose of the study.

**Figure 1:**
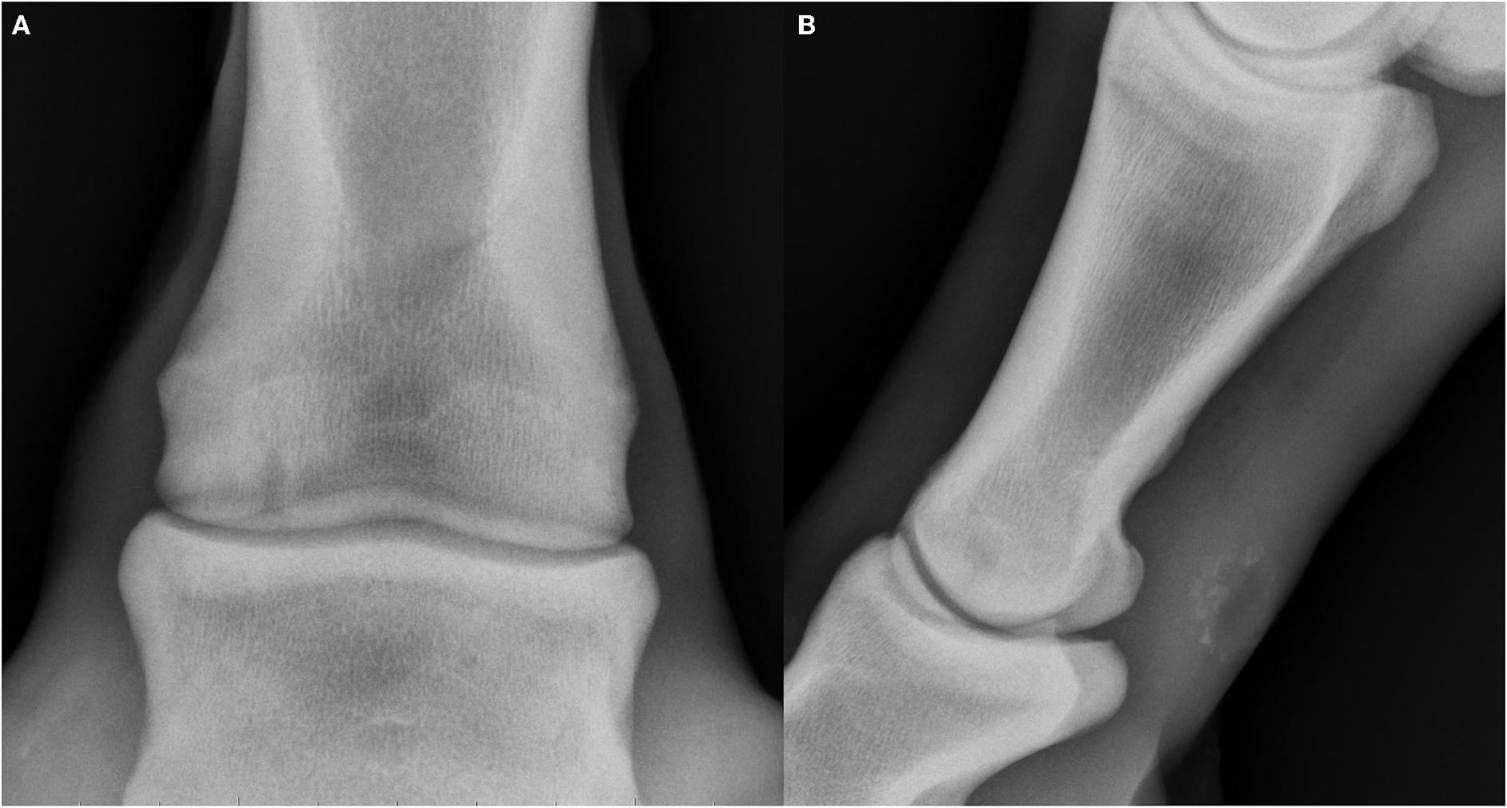
Radiographs of subchondral lucencies at the distal aspect of the proximal phalanx (P1SC), dorsal and lateral are oriented at the left of the images. **(A)** Dorsopalmar projection showing a well-defined, columnar subchondral lucency of the distal aspect of the proximal phalanx with rim of surrounding sclerosis (subtype 3). **(B)** Lateromedial projection showing a well-defined, rounded subchondral lucency of the distal aspect of the proximal phalanx with narrow cloaca extending distally to the joint and rim of surrounding sclerosis (subtype 4).

**Figure 2:**
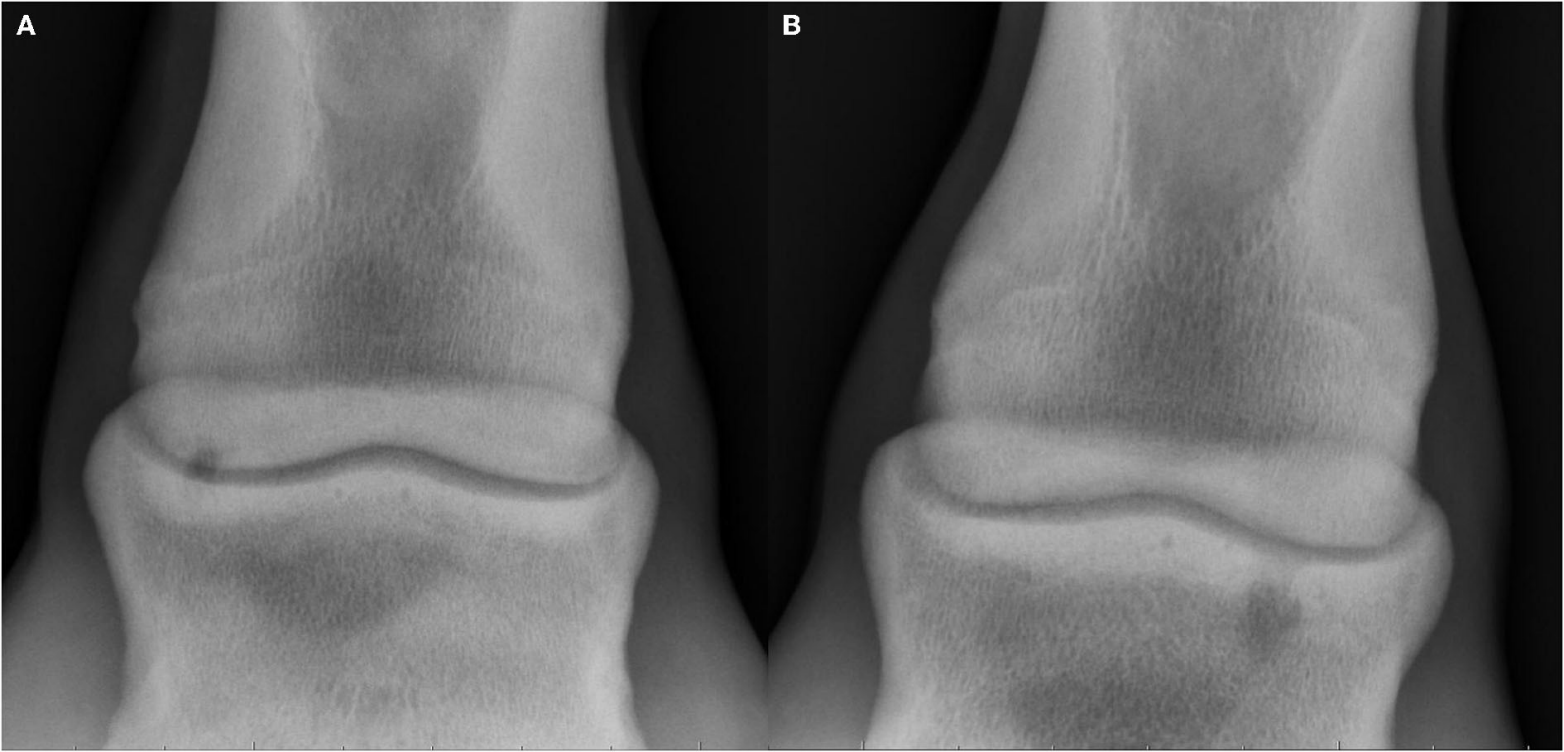
Radiographs of subchondral lucencies at the proximal aspect of the middle phalanx (P2SC), lateral is oriented at the left of the images. **(A)** Dorsopalmar projection showing a well-defined shallow subchondral lucency in the proximolateral aspect of the middle phalanx (subtype 1). **(B)** Dorsoplantar projection showing a well-defined, ovoid subchondral lucency of the proximal aspect of the middle phalanx with surrounding zone of mildly increased sclerosis and a wide ill-defined cloaca extending proximally to the joint (subtype 3).

**Figure 3:**
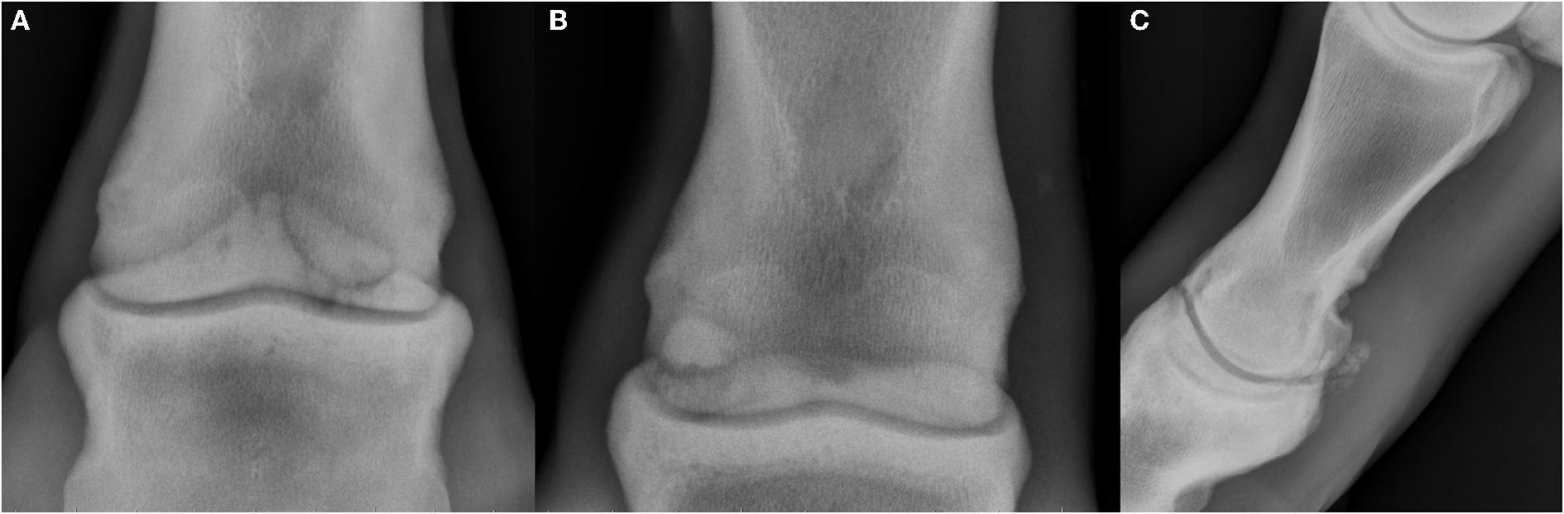
Radiographs showing osteochondral fragmentation of the proximal interphalangeal joint (PIPJ), dorsal and lateral are oriented at the left of the images. **(A)** Dorsopalmar projection showing a large enthesophyte on the proximopalmar aspect of the middle phalanx with large well-defined osteochondral fragment at the palmaromedial aspect. **(B)** Dorsoplantar projection showing a well-defined, smoothly marginated osteochondral fragment at the palmaroproximolateral aspect of the middle phalanx adjacent to a concave defect in the parent bone. **(C)** Lateromedial projection showing numerous poorly marginated osseous fragments at the plantar aspect of the PIPJ with periarticular remodeling which is most severe dorsally.

The digital radiographs of the affected PIPJs of the short-listed horses were reviewed by the first author (J. Faulkner) using DICOM files and digital imaging software (eFILM, Merge Healthcare) and those which met the inclusion criteria were collected for the case group. Inclusion criteria for a subchondral lucency case was any focal decrease in radiopacity of the subchondral bone plate with or without lucency of the immediately adjacent trabecular bone (Figures 1, 2). The inclusion criterium for osteochondral fragment cases was one or more well defined, rounded mineral opacities associated with the joint (Figure 3). Horses were excluded from the case group if fragments or lucencies were identified outside the area of interest (e.g., diaphyseal location). If the radiographic report referred to “possible osteochondral fragmentation” but the radiographic appearance was equivocal the case was excluded from the study. For each case the microchip number, dam, sire, date of birth, sex, examination date, sale and lot number were recorded.

### Radiographic lesions

Lesions were categorized into three lesion types: subchondral lucencies of the proximal phalanx (P1SC), subchondral lucencies of the middle phalanx (P2SC) and osteochondral fragmentation (OCF).

**Figure 4:**
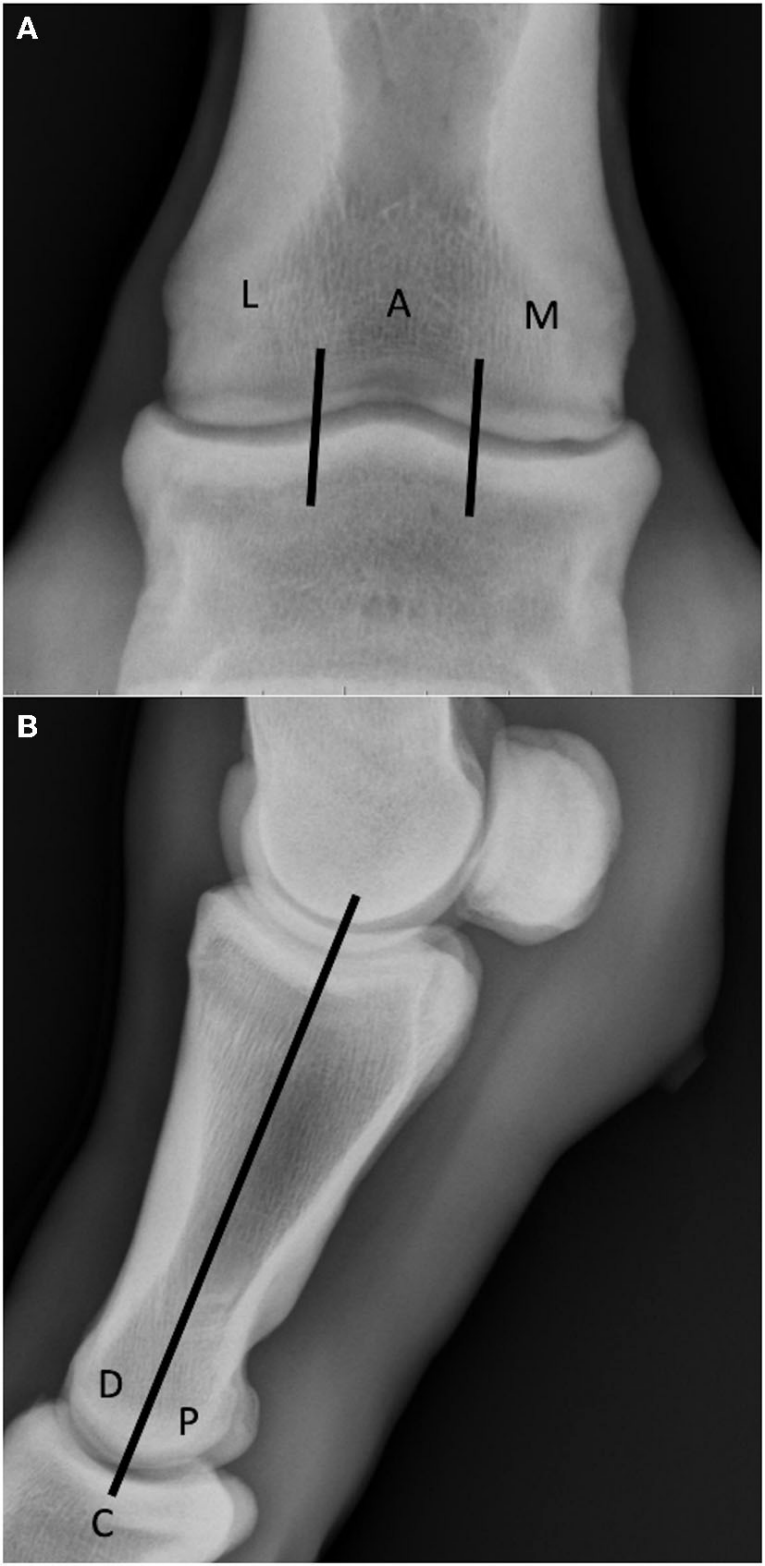
(A) Lesion location on the DP radiograph was determined by dividing the joint into three equally sectioned zones: lateral (L), axial **(A)** and medial (M). Note the well-defined shallow concave subchondral defect in the distomedial aspect of the proximal phalanx (P1SC subtype 1). **(B)** Lesion location on the LM radiograph was performed by dividing the articular surfaces into two equally sectioned zones: dorsal (D) and palmar/plantar (P), and a central region at their border (C).

**Figure 5:**
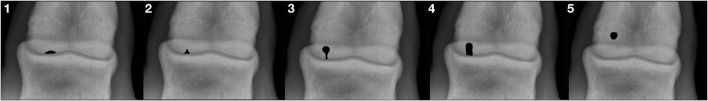
Categorization of subchondral lucency subtypes: **(1)** Well defined shallow dome shaped subchondral defect (<3 mm height), **(2)** Short well defined triangular or fissure-like defects (<3 mm height and width), **(3)** Lollipop or mushroom shape, round or oval cystic lucency extending to the articular surface, with narrow cloaca, **(4)** Columnar shape extending to a large articular surface deficit and **(5)** Lucency without an articular deficit and no radiographic evidence of a cloaca in the subchondral bone plate.

Location of the lucencies and fragments was recorded according to limb (RF, LF, RH, LH) and to three zones on the DP projections (lateral, axial or medial) and three regions on the lateromedial projection (dorsal, central or palmar/plantar; Figure 4). Subchondral lucencies were further subcategorized into five subtypes according to the shape they most closely resembled (Figure 5).

For each lesion, the dimensions (maximum length, L; maximum width, W) were measured with electronic calipers on the DP projection. These measurements were used to calculate elliptical area of best fit (π × L/2 × W/2, mm^2^). If the lesion was not visible on the DP projection, the measurement was made on an alternative view where it was best visualized, such as an oblique projection. Focus-plate distance and plate positioning were approximately standardized during acquisition therefore the effects of magnification were assumed to be negligible.

Periarticular remodeling of the PIPJ was subjectively graded as none (smooth rounded periarticular margins), mild (small pointy periarticular bone formation consistent with osteophytosis, not considered clinically relevant), moderate (moderate amount of pointy periarticular bone formation, determined to be of likely clinical relevance) or severe (large amount of new bone formation, with or without subchondral lucency, suspected to be of definite clinical significance). Any concurrent radiographic abnormalities of other joints which were attributed a moderate or high risk with respect to racing performance by the author of the report were also recorded. Risk analysis had been performed according to existing literature which evaluated effects of the radiographic lesions on future racing performance (11–15). Findings of low or mild risk were those are unlikely to become clinically significant or affect performance. Moderate risk lesion were ones that had a reasonable chance (i.e., 50%) of causing a clinical problem and impacting performance. High risk lesions were ones that were considered likely to become clinical and impact performance.

### Control groups

Two control groups were used. The first, Control Group A, comprised of maternal siblings of the case horses which were identified through the Australian Studbook (http://www.studbook.org.au) and only offspring which were bred and born in Australia were included. Foals registered as born deceased or deceased after birth were excluded. Horses were excluded from Control Group A if they were born after 2014. This date was chosen to ensure that all siblings had the opportunity to achieve a full racing career. A recent study of Australian Thoroughbreds born in 2010 showed that the median age of retirement from racing was 5 years of age (17) and a value double this was chosen. Upon examination of the horses included in Control Group A the horse with the longest racing career retired at 10 years of age, confirming this period to be appropriate. Control Group B consisted of yearlings which had pre-sales repository radiographs obtained by Randwick Equine Center between December 2007 and March 2011 and did not have a radiographically visible PIPJ JOC. To acquire a similar sample size to Control Group A, a similar sized group of yearlings were randomly selected (“Rand()” function in Microsoft Excel) using the same seasonal proportions.

### Outcomes

#### Sales data

Yearling sale price was obtained from an Australian public auction database (https://salesresults.bloodstock.com.au). The auction results were recorded as either (a) the agreed sale price (b) did not meet reserve with reserve price noted, (c) withdrawn from sale or (d) not entered for public auction. For the case group, sales price was preferentially recorded from the auction to which the repository set was submitted. If a case horse did not meet the reserve price or was withdrawn from that auction but sold at a future yearling auction, the amount from the subsequent auction was used instead, with the assumption that there may be an unrelated reason for the withdrawal or failure meet the reserve price at the first sale and that the radiographic appearance of the lesion/s would not change substantially in the relatively short period between the different yearling sales (1). If a case horse was passed in at multiple auctions due to bids not meeting the reserve price and therefore failing to sell, the lowest reserve price was recorded. This approach was utilized to get the best estimation of the value of the horse since the reserve price is the proposed minimal monetary worth set by the seller and the bids are a representation of how much buyers consider the horse is worth. For Control Group A and B, if the horse was sold more than once at a public auction as a yearling, the highest value was recorded. In this situation the assumption was again made that the radiographic appearance of the PIPJ JOC lesion/s would not change in the short period between the different sales and that there may have been another unrelated reason present for the decreased sale price at the first sale.

#### Racing performance

Registered race names for the horses were obtained from the Australian Studbook (http://www.studbook.org.au). For horses which went on to race in Australia, racing performance data was collected from the Racing Australia online database (www.racingaustralia.com.au/horse). For those exported, racing performance data was collected from Racing and Sports Pty Ltd (www.racingandsports.com). For each horse the number of race starts, number of wins, second and third placings and total career earnings in Australian dollars (A$) were recorded. Performance data was included for the full racing career of the horses (data collection in February and March 2022).

### Data analysis

The case group was compared with control groups A and B and the lesion types (P1SC, P2SC, OCF) were compared within the case group with respect to the proportion of horses which successfully sold when entered for sale at the public auctions and the proportion of horses which started at least one race, with the use of Fisher's Exact Test.

The case group was compared with Control Group A with respect to sales price data (including only sold horses), number of races, number of places (first, second or third), total career prize money and average prize money per race using a mixed effects model with dam as random effect and population (cases vs. maternal siblings) as fixed effect with normally distributed error term. For the comparison between the case group and Control Group B the same analyses were done but excluding the dam effect.

Lesion types (P1SC, P2SC, OCF), location (limb, region on DP and LM radiographs), lesion size (area in mm^2^) and presence of concurrent radiographic abnormalities were compared within the case group with respect to sales price data (including only sold horses), number of races, number of places (first, second or third), total career prize money and average prize money per race using a fixed effects model with normally distributed error term. If horses had more than one lesion present in different locations they were excluded from the relevant analysis. A summed area of lesions was used for horses with more than one lesion in the size analysis.

Two types of sensitivity analyses were performed, firstly with adjustment factors of sex and season (birth year) and secondly by including the horses which failed to sell by including the reserve prices as a proxy value.

Statistical analysis was performed using Sas Version 6.4. Statistical significance was set at *p* ≤ 0.05 and a trend defined as *p* ≤ 0.1.

## Results

### Descriptive findings

#### Case group

Repository radiographs for 1,098 yearlings were acquired during the study period and 6.3%, (*N* = 69; 32 male, 37 female) had at least one radiographically apparent PIPJ JOC lesion. One horse was excluded due to the presence of an equivocal lesion described in the radiographic report as possible osteochondral fragmentation. Fifty-three horses (4.8%) had P1SC, seven (0.6%) had P2SC, and nine (0.8%) had OCF. One limb was affected in 58 horses, two limbs in 10 horses (OCF *N* = 2, P1 SC *N* = 8) and three limbs in one horse (P1SC *N* = 1). In all cases where more than one limb was affected, the same lesion type was present in all the affected limbs. The majority of horses had one lesion per limb (67/69). Two horses had multiple PIPJ lesions in one limb (P1 SC *N* = 2, one with one limb affected and the other with two limbs affected). In total there were 84 PIPJ JOC identified (P1SC *n* = 66, P2SC *n* = 7, OCF *n* = 11). The summary data for variability in lesion location, size and shape is shown in Tables 1, 2 and degree of joint modeling associated with the proximal interphalangeal radiographic lesions in Table 3.

**Table 1:** Number, location and size of PIPJ JOC for horses in the case group.

	**All PIPJ JOC**	**P1SC**	**P2SC**	**OCF**
Number of horses	69	53	7	9
Number of lesions	84	66	7	11
*RF*	23	20	1	2
*LF*	21	18	2	1
*RH*	19	13	2	4
*LH*	21	15	2	4
*Lateral*	20	15	3	2
*Axial*	34	29	3	2
*Medial*	25	21	1	3
*Axial and medial*	3	1	0	2
*Lateral and medial*	1	0	0	1
*Lateral, axial and medial*	1	0	0	1
*Dorsal*	9	6	0	3
*Central*	7	7	0	0
*Palmar/plantar*	25	17	0	8
*Not identified on LM radiograph*	43	36	7	0
*Mean area (mm^2^)*	27.0	13.1	18.0	116.6
*Area range (min–max, mm^2^)*	0.8–534.1	0.8–51.8	7.9–42.4	31.4–534.1

**Table 2:** PIPJ JOC lesions by type and subtype.

**Lesion subtype**	**1**	**2**	**3**	**4**	**5**	**Total**
P1SC	22	6	20	11	7	66
P2SC	3	1	3	0	0	7
OCF	n/a	n/a	n/a	n/a	n/a	11

**Table 3:** PIPJ JOC lesion type by degree of joint modeling.

**Grade of joint modeling**	**P1SC**	**P2SC**	**OCF**	**All PIPJ JOC**
*None*	39	2	4	45
*Mild*	22	5	4	31
*Moderate*	2	0	2	4
*Severe*	0	0	1	1
Total number of joints	63	7	11	81

Eleven horses (P1SC *N* = 7, P2SC *N* = 2, OCF *N* = 2) had concurrent radiographic lesions present in other joints which were considered to have overall moderate (*N* = 7) or severe (*N* = 4) risk for negative impact on racing performance. The lesions included proximal interphalangeal joint modeling in another limb unaffected by PIPJ JOC (2); fetlock osteochondral fragmentation (3), sagittal ridge osteochondrosis (1), distal third metacarpal subchondral bone cyst (3), supracondylar lysis (1), basilar proximal sesamoid fragmentation (1), osseous modeling of the sesamoid bones (3); metacarpal osseous remodeling associated with a wound (1); carpal subchondral lucency and modeling (2); lateral femoral trochlear ridge osteochondrosis (2) and medial femoral condyle osseous cyst like lesion (3). The remainder of horses (58/69) had either no other radiographic lesions in other regions or radiographic lesions which were classified as low or mild risk.

#### Control groups

There were 397 maternal siblings which fulfilled the criteria for inclusion in Control Group A. To acquire a similar sample size for Control Group B, 400 yearlings were randomly selected from the horses without PIPJ lesions on repository radiographs acquired in the same period. Nine horses were excluded due to not being born in Australia resulting in 391 horses in Control Group B.

### Sales

The proportion sold at public auction was not significantly different between the case group (79.7%) and Control Group A and B (86.53 and 84.1%, respectively; *p* > 0.1). The proportion of yearlings sold at public auction was significantly lower for OCF (44.4%) compared with P1SC and P2SC (84.9 and 85.9%, respectively; *p* ≤ 0.05).

There was no significant difference between the case group and Control Group A and B for sales price, both without and with an adjustment for sex and season and including the reserve prices as a proxy for those horses which did not meet the reserve (*p* > 0.1; Table 4 and Supplementary Table 1). Similarly, there was no significant difference between the JOC lesion types for sales price (*p* > 0.1).

**Table 4:** Means *(standard error of the mean)* of the case group and control groups for sales and racing performance and their mean differences *(standard error of the mean)*.

	**Case group**	**Control group**	**Difference**
**Sales price (A$)**			
Case group and control group A	228,619 (38,593)	191,861 (27,777)	36,759 (36,668)
Case group and control group B	216,109 (37,569)	208,409 (15,361)	7,700 (40,587)
**Sales price incl. reserve price proxy (A$)**			
Case group and control group A	214,128 (33,019)	174,931 (22,946)	39,198 (33,092)
Case group and control group B	214,567 (33,108)	207,497 (14,013)	7,070 (35,951)
**Number of races**			
Case group and control group A	14.38 (2.01)	15.50 (0.85)	−1.12 (2.17)
Case group and control group B	14.38 (2.08)	17.12 (0.87)	−2.75 (2.26)
**Number of places**			
Case group and control group A	4.78 (0.72)	5.36 (0.31)	−0.58 (0.78)
Case group and control group B	4.78 (0.78)	5.96 (0.33)	−1.17 (0.85)
**Total career prize money (A$)**			
Case group and control group A	43,347 (21,608)	82,112 (9,345)	−38,765 (23,296)^*^
Case group and control group B	43,347 (19,804)	83,523 (8,319)	−40,176 (21,480)^*^
**Average prize money per race (A$)**			
Case group and control group A	2,417 (981)	4,292 (437)	−1,875 (1,051)^*^
Case group and control group B	2,417 (889)	3,842 (373)	−1,426 (964)

Control Group A comparison values are derived from mixed effects model. Control Group B comparison values are derived from fixed effects model.

* Indicates p ≤ 0.1.

**Figure 6:**
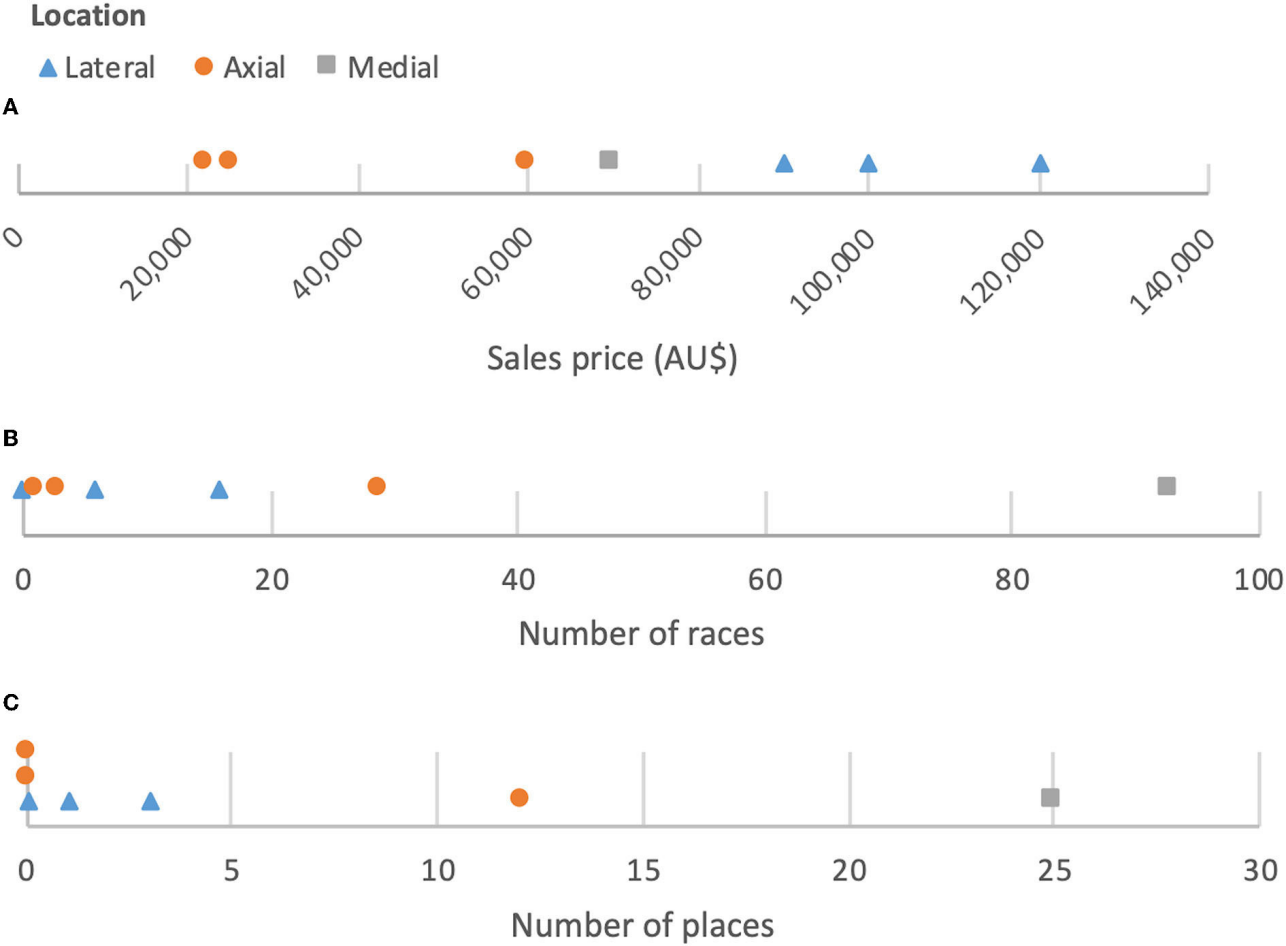
Line plots showing distribution of P2SC location on dorsopalmar/plantar radiograph by **(A)** sales price, **(B)** number of races, and **(C)** number of places (*N* = 7).

When comparing lesion characteristics (location and size) with respect to sales price, the location of P2SC on a DP radiograph was associated with a significant difference in mean sale price with those with axial lesions on average selling for less (A$ 23,400) than laterally and medially located lesions [A$ 103,333 and A$ 70,000, respectively; *p* ≤ 0.05]. Distribution of the data is shown in Figure 6A. The difference remained significant after the inclusion of reserve prices as proxies for sale value (Axial - A$ 35,667, Lateral A$ 103,333, Medial A$ 70,000; *p* ≤ 0.05). The sample size of horses within this group is small (*N* = 7) so this should be carefully interpreted. There were no significant associations between the any of the other lesion characteristics and sales price (*p* > 0.1).

Three of four horses with moderate joint remodeling sold successfully at the sale (75%, mean sale price A$ 60,000). The horse with severe joint remodeling did not successfully sell due to failure to meet the reserve price. The presence of concurrent high or moderate risk radiographic abnormalities of other regions was associated with a significantly lower sale price (absence of risk lesion- A$ 253,589, presence of risk lesion- A$ 47,450; *p* ≤ 0.05). The difference remained statistically significant when reserve prices were included as proxies for sale price (absence of risk lesion - A$ 245,563, presence of risk lesion- A$ 56,773; *p* ≤ 0.05).

### Racing performance

The proportion of horses which started at least one race was not significantly different between the case group (79.7%) and Control Group A and B (80.9 and 85.7%, respectively; *p* > 0.1). The proportion of horses which started at least one race was not significantly different between lesion types, with 83.0, 85.7, and 55.5% of horses with P1SC, P2SC, and OCF, respectively making it to the racetrack (*p* > 0.1).

There was a trend toward lower total career prize money earned by the horses of the case group when compared to both Control Group A and B (*p* = 0.0657 and *p* = 0.0898, respectively; Table 4) which persisted following adjustment for sex and season (Supplementary Table 1). When evaluating the average prize money per race there was a trend toward a lower value for the case group when compared with Control Group A (*p* = 0.0753; Table 4). The difference became more insignificant once adjustments for sex and season were considered (*p* > 0.1; Supplementary Table 1). There were no significant differences between the case group and Control Group B for the average prize money per race (*p* > 0.1). No significant differences were found between the case group and both control groups A and B for the number of races in the career or number of places (*p* > 0.1). There were no significant differences between the JOC lesions types for any measure of racing performance (*p* > 0.1).

The location zone of the lesion on the LM radiograph for all JOC combined was associated with a significant difference in average prize money per race, with those with lesions in a central location on average earning a higher amount (A$ 4,298.21), than those in a dorsal or palmar/plantar location (A$ 1,950.46 and A$ 1,365.58, respectively; *p* ≤ 0.05). Location zone on the LM radiograph had an association with total career prize money that approached statistical significance, with those with lesions in a central location earning a higher mean amount (A$ 102,465) than those in a dorsal or palmar/plantar location (A$ 35,271 and A$ 24,804, respectively; *p* = 0.0882). The number of places was also close to being significantly different, with those having lesions in a central location having a higher average number of places (9.8), than those in a dorsal or palmar/plantar location (3.1 and 4.4, respectively; *p* = 0.0889). When assessed separately for the lesion types, there was a trend toward a difference in average prize money per race for P1SC depending on location on LM radiograph with those with centrally located lucencies earning more on average (A$ 4,298.21) than those with dorsally or palmar/plantarly located lucencies (A$ 2,514.12 and A$ 1,323.43, respectively; *p* = 0.0865).

The location of P2SC on a DP radiograph was associated with a significant difference in the mean number of races run, with a higher mean number of races run by those which had a lucency in the medial zone (93) than those with a lucency in the axial or lateral zones (11 and 7.3, respectively; *p* ≤ 0.05). Horses with a medially located P2SC also had a statistically significant higher mean number of places (25) than those with an axial or lateral location (4 and 1.3, respectively; *p* ≤ 0.05). Distribution of the data is shown in Figures 6B,C. The sample size of horses within this group is small (*N* = 7) and there is a high performing outlier with a medial location with high number of races (93) and places (25), so this result should be carefully interpreted.

**Figure 7:**
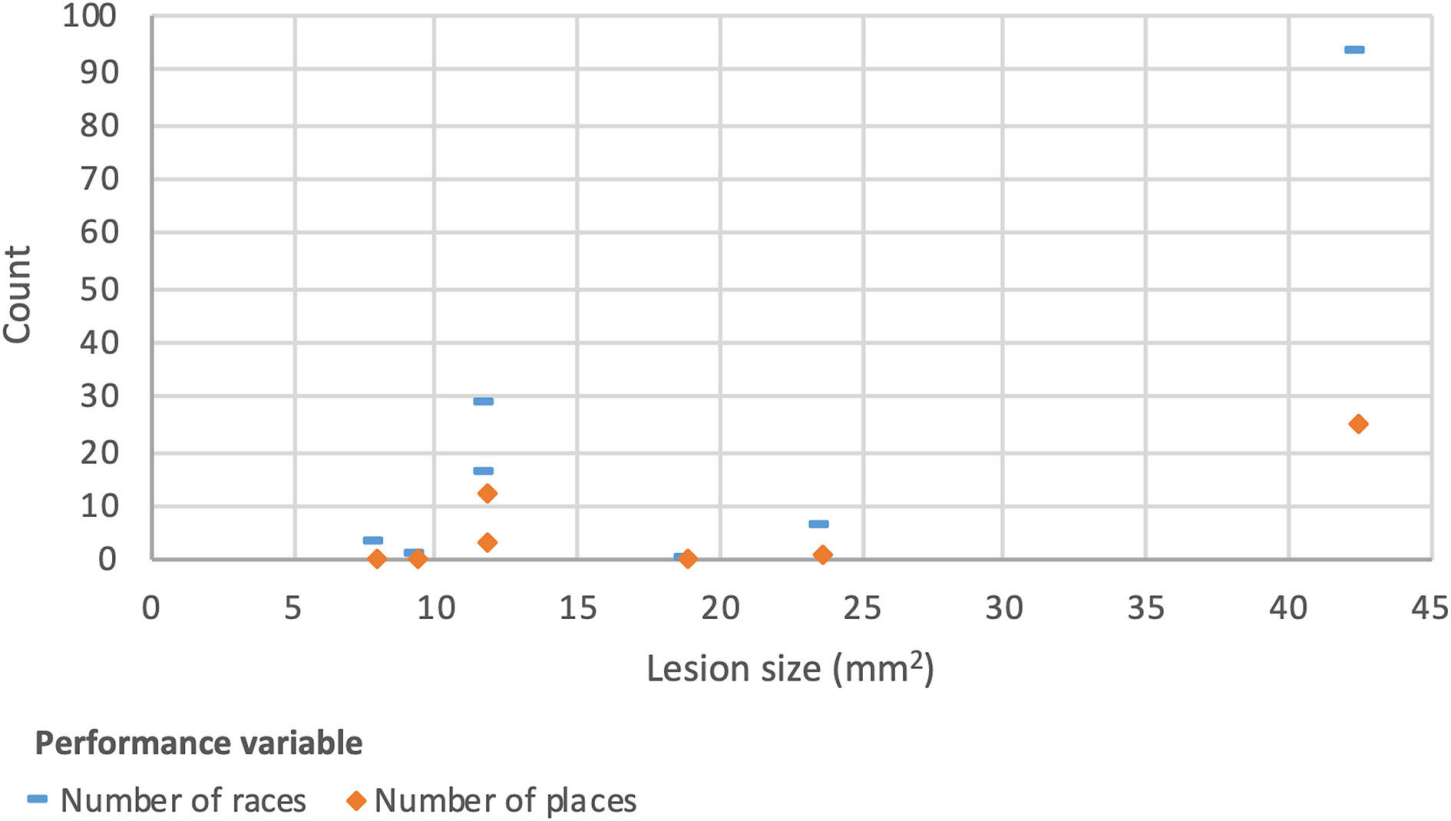
Scatter plot showing distribution of P2SC size by number of races and places (*N* = 7).

The size of P2SC lesions had an association with a significant difference in the number of races run (slope estimate 2.3; *p* ≤ 0.05) and number of places (slope estimate 0.6; *p* ≤ 0.05) with larger lesions performing better on both variables. Distribution of the data is shown in Figure 7. The abovementioned high performing outlier had the largest size (42 mm^2^) so again this result should be carefully interpreted.

**Figure 8:**
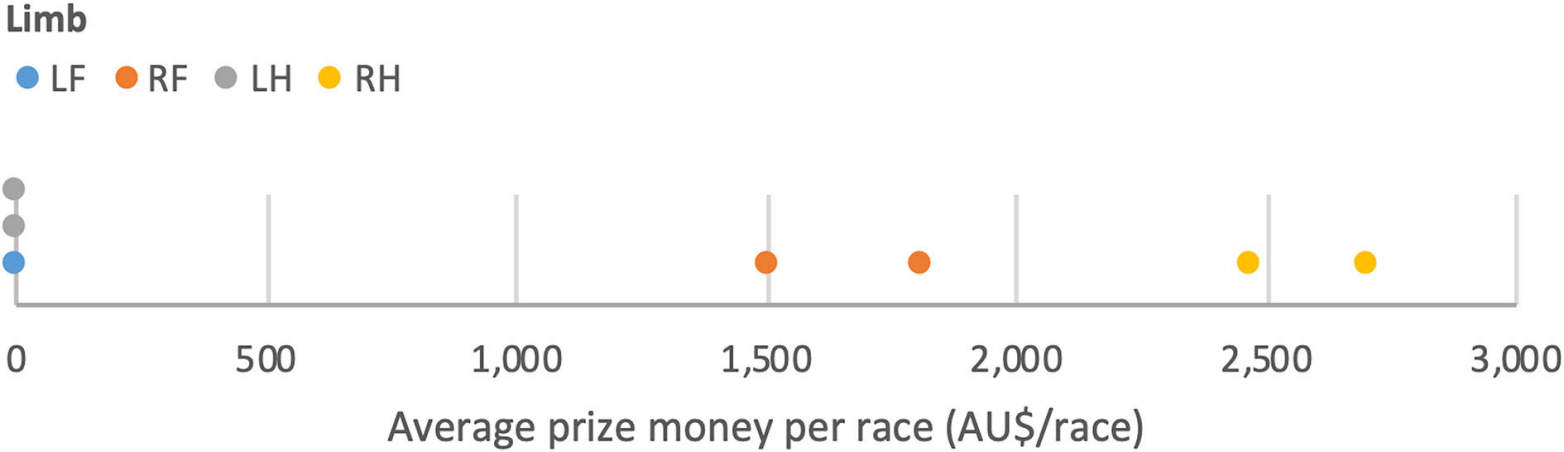
Line plot showing distribution of limb location for OCF by average prize money per race (*N* = 7).

The limb in which an OCF was located was associated with a significant difference in average prize money earned per race with those with OCFs in the LF and LH earning less per race (A$ 0 and A$ 0, respectively) than those with OCFs in the RF and RH (A$ 1,654.62 and A$ 2,585.20, respectively; *p* ≤ 0.05). Distribution of the data is shown in Figure 8. The sample size of horses within this group is small so this result should be interpreted carefully (*N* = 7, as two horses were removed from the OCF group for this analysis due to multiple limb involvement). An association of total career prize money with limb location approached significance for horses with P1SC, with those with lucencies in the RH on average earning more (A$ 106,319) than the other limbs (LF - A$ 44,819, RF - A$ 21,358, LH - A$ 37,066; *p* = 0.0955).

Three of four horses with moderate joint remodeling successfully entered the racing circuit (mean number of races 8, mean total career prizemoney A$ 12,536). The horse with severe joint remodeling did not race. Case horses were less likely to be exported than horses in the control groups (Case horses 5/69 = 7.2%, Control Group A 40/399 = 10%, Control Group B 60/393 = 15.3%). Horses with presence of concurrent radiographic abnormalities of other regions that were attributed a moderate or severe risk for racing had a trend toward earning higher total career prize money (A$ 79,879) compared to the horses with no other radiographic abnormalities or radiographic abnormalities assigned a mild risk (A$ 36,419; *p* = 0.0675).

## Discussion

The reported prevalence of radiographic abnormalities in the PIPJ of other populations of Thoroughbred yearlings has ranged from 1.3% (USA) to 3.96% (France) (5, 15). In this population of Australian Thoroughbred yearlings entered for public auction the prevalence of PIPJ JOC was 6.3%. This higher proportion may be associated with genetic and environmental influences, selection bias for the sales and/or different detection sensitivity (e.g., number of radiographic projections included for review, radiographic quality or differences in reader scrutiny).

Overall, the prevalence of PIPJ abnormalities has been reported to be greater in hindlimbs than forelimbs (2–5, 15). In this population of Australian Thoroughbred yearlings, the proportion of P2SC and OCF was greater in the hindlimbs (57.1 and 72.7%, respectively). However P1SC were more common in forelimbs (57.6%), which is similar to a previous publication examining a young Thoroughbred population in USA which found a higher proportion of distal P1 subchondral bone cysts in the forelimbs than in hindlimbs (5). Converse to our findings in Australian Thoroughbreds, subchondral cystic lesions in the distal aspect of the proximal phalanx were predominantly located in the hindlimb in French Thoroughbreds as well as in French Standardbred trotters and Selle-Français warmbloods (1). There was no evident disadvantage of lesion location in the forelimbs vs. the hindlimbs for racing performance in the current study.

In the current study, P1SC was found in 76.8% of the horses with JOC whereas P2SC and OCF was present in a lower proportion of horses (10 and 13%, respectively). This relatively high prevalence of subchondral lucencies of distal P1 in this study corresponds well with the Thoroughbred population in USA (5). It is also similar to the findings in young horses in France (1) where unilateral juvenile subchondral bone cysts were found to be the second most frequent radiological finding in the PIPJ (6.2%) after proximal physitis of the middle phalanx and where 90% were located in the distal condyle of the proximal phalanx.

The previously reported prevalence of OCF of the PIPJ has ranged from as low as 0.1% in young North American Thoroughbreds (5) and 0.9% in 3–7 year old Hanoverian Warmbloods Stock et al. (3) to as high as OCFs being present in the hindlimbs of 4.56% of Thoroughbred yearlings in France (15). OCFs were present in 0.8% of the horses with repository radiographs in the current study, with lesions located in the hindlimbs of 66% of horses and two of these horses were bilaterally affected.

Consistent with previous literature, axial location of PIPJ JOC lesions was less common than the condylar regions however the relative proportion of subchondral lucencies located in the axial zone was relatively larger in this study compared to previous studies (5). This may be due to population variation or due to differences in inclusion of certain midline lucencies between studies. Some focal midline lucencies projected through distal P1 on a DP radiograph can represent the margins of the palmar or plantar inter-condylar fossa and deemed to be a normal variation in some circumstances (5). Subchondral lucencies of the PIPJ have historically been considered an incidental finding if located in the axial aspect of the distal articular surface of the proximal phalanx, with those on the medial or lateral condyles considered more clinically significant, especially if they communicate with the articular surface (18). These laterally and medially located lesions have been associated with severe degenerative joint disease in some young horses (8, 9). However a conflicting study abstract showed that medial and lateral subchondral lucencies did not have an effect on racing performance and that large lucencies in the axial portion of the pastern negatively affected performance (6). In the study only axial lesions which were subjectively classified as large in size and visible on two views were recorded on the radiographic report whereas the medial and laterally located lesions were recorded in all circumstances. The authors of the study proposed that the horses with midline lucencies that were included in the analysis represented those with more severe lesions, and therefore may have been more likely to become clinically relevant than smaller medial and lateral lesions. In the current study, there was no significant difference in performance for horses with P1SC at the medial, axial or lateral aspects of the articular surface. Lack of association with decreased performance may be due to small or nonexistent joint communication as osseous cyst like lesions deep within bones are reported to be less likely to be associated with lameness than articular lesions (19).

In the current study, a JOC lesion in a dorsal or palmar/plantar location on the LM radiograph was associated with lower average prize money per race (*p* ≤ 0.05), total prize money (*p* ≤ 0.1) and number of places (*p* ≤ 0.1) than those located centrally. For P1SC, a central location of a lesion was associated with a larger average prize money per race (*p* ≤ 0.1). All centrally located lesions were P1SC (7/7, 100%), with the majority located in an axial location (6/7, 85.7%). It is proposed that P1SC lesions with location in the axial and central location zones may avoid negative impacts on performance as it this is a low contact region of the joint which experiences relatively low stress.

When assessing location of PIPJ JOC lesion types further, it was found that axial P2SC were sold for significantly less than P2SC lesions located medially or laterally (*p* ≤ 0.05). Larger P2SC and those with medially located lesions had a higher number of races run and places (*p* ≤ 0.05). Due to the low sample group size for P2SC and the presence of a highly performing outlier, the results must be conservatively interpreted, however from Graphs 8, 9 and 10 it can be appreciated that there were horses with lesions of a range of sizes and in all regions on the DP radiograph which performed well over numerous occasions and some large PIPJ JOC may be compatible with a high level racing career. Similarly, for OCF although the limb in which the lesion was located was associated with a significant difference on average prize money earned per race (*p* ≤ 0.05) this result needs to be interpreted with care. It is feasible that those with left sided lesions earnt significantly less per race run due to racing direction.

When assessing all horses in the case group in comparison with the control groups, the horses with JOC had a decreased total career prize money (*p* ≤ 0.1) and average prize money per race (*p* ≤ 0.1 when compared with Control Group A) but there was no significant difference between the case group and control groups for the number of races in the career or number of places. Therefore, while the presence of these lesions showed a trend toward decreased racing performance it was not the situation for all variables investigated. Based on these findings it can be suggested that veterinarians reading repository radiographs should not attribute a high risk to these lesions, although concurrent severe early osteoarthritic changes should be considered as the one horse with severe joint remodeling in this study did not successfully sell or race. Sales results were hypothesized to mirror the risk to racing performance and while fewer yearlings with PIPJ JOC successfully sold at auction it was shown that overall there was no significant differences detected in sale price between the case group and control groups indicating that this is probably the case.

When assessing the JOC lesion types separately there was a higher proportion of horses with OCF (55%) which did not race, when compared with P1SC (83%) and P2SC (86%) and those in the control groups (A - 73%, B - 75%) however this was not statistically significant (*p* > 0.1). The lower proportion of horses with OCF which sold at the auctions (44.4%), when compared with those with P1SC (84.9%) and P2SC (85.9%) and those in the control groups (A - 86.5%, B - 84.1%) was statistically significant (*p* ≤ 0.05). Interestingly, failing to sell at the auction did not preclude horses from racing. For all horses with JOC there were 12 which failed to sell and eight of these raced. For those with OCF one of the four horses which failed to sell went on to make it to the racetrack.

The etiology of osteochondral fragmentation is deemed by many to be multifactorial and can be of developmental and traumatic origin (19, 20). Surgical findings have indicated that fragments located at the dorsomedial and dorsolateral aspect of the PIPJ are often avulsion fractures at the origin of the collateral ligament of the distal sesamoid (navicular) bone and it is suggested that a traumatic etiology is more likely when accompanied by an aggressive proliferative reaction even when bilaterally present (21). In the Australian Thoroughbreds included in this study there were two with dorsomedial OCF and both had moderate associated PIPJ modeling. Of these horses, one failed to sell at the auction and did not race and the other sold below the average sale price and fell below average for all performance variables investigated.

Osteochondral fragments located at the palmar/plantar aspect of the PIPJ have shown varying amounts of articular involvement at arthroscopy (20). In cases whether the fragments are located in the insertions of the SDFT, abaxial and axial palmar ligaments and straight distal sesamoidean ligament they are thought to be more likely due to trauma (5, 22). Since decreased racing performance is associated with yearlings with enthesophyte formation at the base of the proximal sesamoid bones in the hindlimb (11) it has been proposed that large enthesophyte formation and/or fragmentation in the region of soft tissue attachments of the middle scutum may also result in performance limitation (5). In the Australian Thoroughbreds included in this study there were six horses with palmar/plantar OCF. Only three were sold and four raced, all winning less than prize money over their careers than the control group averages.

Regarding the limitations of the study, the small sample size could have been increased by including horses born up until 2016, and considering racing performance up to the median age of retirement of 5 years (17). This would have improved some constraints of the statistical analysis however would have not been a complete representation of the racing results for the whole career of all horses. Other limitations of the study include the selection bias of using a sub-population of Australian Thoroughbreds from certain studs all located in New South Wales and the inclusion of only yearlings which have been selected to go to public sale. The absence of a physical or lameness examination and lack of clinical information about other performance limiting conditions is also a restriction in the interpretation of the results, as is the lack of information about the management or treatment of the horses following sale since some PIPJ lesions may be management responsive (for example delayed breaking in and weight control) or surgically treated (9, 20, 22–28).

The radiographic evolution of the PIPJ JOC lesions identified has not been assessed in the current study. It has been shown that juvenile osseous cyst-like lesion identified at the age of 6 months can disappear or improve in appearance by 18 months (1). Additionally in the same study 40% of RFs identified at 18 months were not seen at 6 months (1). The subchondral lucencies identified in the current study were not graded for degree of lucency, and some may have been in the process of filling in and resolving. The Type 2 subtype of lucency described here has not previously been reported and is postulated to indicate the remnant or originating point of a Type 3 lesion.

Further shortcomings of the use of racing results to assess the clinical relevance of a radiographic lesion include the wide ranges in outcome data such as number of starts and money earned. It was presumed that all horses in the study raced on the flat as this is the major form of Thoroughbred racing in Australia however this was not corroborated from the racing data. The study is also limited by the lack of information available for the reason horses may have retired early and had a short racing career (e.g., breeding, respiratory disease, other musculoskeletal injury). Racing results from horses which raced internationally were included and may have dissimilar racing circumstances in different locations, for example difference in length of career or prize money pool available. A smaller proportion of horses with PIPJ JOC were exported than for horses in the control groups, which may be due to the presence of the lesions or other reasons. It is possible that the trend of case horses earning lower career prize money and lower prize money per race than the control groups (*p* ≤ 0.1) may be partially explained by a higher proportion of control group horses being exported to countries where nominal earnings are higher, such as Hong Kong and Japan (29). Furthermore, the performance results of this study cannot be directly transferred to horses of other breeds and those which are used in other disciplines as racehorses have relatively short and intense athletic careers.

The use of two types of control groups was employed to try to overcome limitations in previous research where only maternal siblings were used for the control group. However, the use of maternal siblings does have the benefit of comparing to horses of similar bloodlines, which the Control Group B in this study was lacking. Although yearling sales prices have been shown to be related to a sire's stud fee and progeny (30), the effect of sire was not adjusted for in this study because firstly osteochondrosis has a genetic component, and secondly to avoid problems in the analysis and interpretation since there were numerous sires which had only one or low numbers of offspring.

Concurrent radiographic abnormalities of other regions which were attributed a high or moderate risk to racing were evaluated to determine concurrent effects they may have on the data. Interestingly the presence of these lesions in the control group was associated with a significantly lower sale price (*p* ≤ 0.05), but a trend toward a higher total career prize money (*p* ≤ 0.1). It could be hypothesized that the management of the horses with high and moderate risk radiographic lesions was altered and had a benefit on performance, however further discussion of these findings is outside of the scope of the article.

Radiographs were initially read by one experienced clinician and the radiographs of reports listing a PIPJ JOC lesion were verified by the primary author. However, the radiographs for the reports which did not list a PIPJ JOC lesion were not reviewed to ensure that none had been overlooked. As the radiographs are not centered on PIPJ the joint is obliquely projected and some lesions can be difficult or impossible to visualize. P2SC were not identified in any LM radiographs, highlighting the importance of acquiring and examining the DP projection for identification of these lesions. Additionally, the obliquity of the PIPJ on the radiographs can make it difficult to establish whether there is articular involvement.

## Conclusion

The prevalence of PIPJ JOC in the group of Australian Thoroughbred yearlings examined is higher than previously reported in other populations and the horses with PIPJ JOC had a trend toward a poorer racing performance for the variables of total career prize money and prize money per race. Horses with dorsally and palmar/plantarly located lesions on the LM projection performed significantly worse than those with centrally located lesions. A significantly lower proportion of horses with OCF were successfully sold at public auction and a lower proportion made it to the racetrack to race, although this was not statistically significant. These findings should be considered when reviewing radiographs of Thoroughbred yearlings.

## Data availability statement

The raw data supporting the conclusions of this article will be made available by the authors, without undue reservation.

## Ethics statement

Ethical review and approval was not required for the animal study because the study is retrospective in nature and the data is publicly available. Written informed consent for participation was not obtained from the owners because the study is retrospective in nature and the data is publicly available.

## Author contributions

JF and CO'S conceived the study design. JF performed the data collection. LD and JF analyzed the data. JF, KV, LD, and CO'S contributed to the data interpretation, manuscript preparation, and approved the submitted version.

## Conflict of interest

The authors declare that the research was conducted in the absence of any commercial or financial relationships that could be construed as a potential conflict of interest.

## Publisher's note

All claims expressed in this article are solely those of the authors and do not necessarily represent those of their affiliated organizations, or those of the publisher, the editors and the reviewers. Any product that may be evaluated in this article, or claim that may be made by its manufacturer, is not guaranteed or endorsed by the publisher.
