# Supplementary material for: Radiographic prevalence of juvenile osteochondral conditions of the proximal interphalangeal joint of Australian Thoroughbred racehorse yearlings and associations with sales results and race performance
Source: Front Vet Sci. 2022 Oct 10;9:988826. doi: 10.3389/fvets.2022.988826 (PMC9589099; doi:10.3389/fvets.2022.988826)
Supplement: Supplementary file 1 [file Table_1.docx]

Supplementary Material

**Supplementary Table 1.** Means *(standard error of the mean)* of the case group and control groups for sales and racing performance and their mean differences *(standard error of the mean)*, adjusted for effect of sex and season. Control Group A comparison values are derived from mixed effects model. Control Group B comparison values are derived from fixed effects model. ***** indicates p ≤ 0.1

|  | **Case Group** | **Control Group** | **Difference** |
| --- | --- | --- | --- |
| **Sales price (A$)** |  |  |  |
| *Case Group and Control Group A* | 152,078 *(50,975)* | 165,498 *(34,615)* | -13,420 *(43,279)* |
| *Case Group and Control Group B* | 203,789 *(37,531)* | 201,119 *(16,346)* | 2,670 *(39,991)* |
| **Sales price, incl. reserve price proxy (A$)** |  |  |  |
| *Case Group and Control Group A* | 148,857 *(45,369)* | 157,548 *(30,348)* | -8,691 *(38,746)* |
| *Case Group and Control Group B* | 204,936 *(32,815)* | 198,223 *(14,880)* | 6,714 *(35,289)* |
| **Number of races** |  |  |  |
| *Case Group and Control Group A* | 14.68 *(2.70)* | 14.63 *(1.39)* | 0.05 *(2.48)* |
| *Case Group and Control Group B* | 14.46 *(1.98)* | 16.25 *(0.88)* | -1.78 *(2.13)* |
| **Number of podium finishes** |  |  |  |
| *Case Group and Control Group A* | 4.94 *(0.99)* | 5.41 *(0.52)* | -0.47 *(0.90)* |
| *Case Group and Control Group B* | 4.81 *(0.76)* | 5.71 *(0.34)* | -0.90 *(0.82)* |
| **Total career prize money (A$)** |  |  |  |
| *Case Group and Control Group A* | **41,281*(29,997)*** | **91,768 *(15,877)*** | **-50,486 *(27,352)* *** |
| *Case Group and Control Group B* | **45,668 *(19,788)*** | **81,806 *(8,840)*** | **-36,138 *(21,256)* *** |
| **Average prize money per race (A$)** |  |  |  |
| *Case Group and Control Group A* | 3,282 *(1,363)* | 5,265 *(743)* | -1,983 *(1230)* |
| *Case Group and Control Group B* | 2,564 *(899)* | 3,929 *(402)* | -1,365 *(966)* |
