# Supplementary material for: Radiographic prevalence of juvenile osteochondral conditions of the proximal interphalangeal joint of Australian Thoroughbred racehorse yearlings and associations with sales results and race performance
Source: Front Vet Sci. 2022 Oct 10;9:988826. doi: 10.3389/fvets.2022.988826 (PMC9589099; doi:10.3389/fvets.2022.988826)
Supplement: Supplementary file 2 [file Table_2.docx]

Supplementary Material

**Supplementary Table 2.** PIPJ JOC location on lateromedial radiograph by lesion type and location on dorsopalmar/plantar radiograph.

|  | Central | Dorsal | Palmar/plantar |
| --- | --- | --- | --- |
| Total | 7 | 9 | 25 |
| P1SC | 7 | 6 | 17 |
| P2SC | 0 | 0 | 0 |
| OCF | 0 | 3 | 8 |
| Medial | 0 | 2 | 10 |
| Axial | 6 | 7 | 3 |
| Lateral | 1 | 0 | 8 |
| Axial and medial | 0 | 0 | 2 |
| Lateral and medial | 0 | 0 | 1 |
| Lateral, axial and medial | 0 | 0 | 1 |
